# Optimal Care Pathways for People with Lung Cancer- a Scoping Review of the Literature

**DOI:** 10.5334/ijic.5438

**Published:** 2020-09-28

**Authors:** Zulfiquer Otty, Amy Brown, Sabe Sabesan, Rebecca Evans, Sarah Larkins

**Affiliations:** 1 Townsville Cancer Centre, Townsville University Hospital, Townsville, QLD, AU; 2 College of Medicine & Dentistry, James Cook University, Townsville, QLD, AU; 3 Australian Institute of Tropical Health and Medicine, James Cook University, Townsville, QLD, AU

**Keywords:** integrated care, lung cancer, care pathways, scoping review

## Abstract

**Introduction:**

Much of the existing work around implementation of cancer optimal care pathways (OCP) has either focused exclusively on the clinical elements of care or has targeted individual stages in the cancer trajectory, rather than using a patient-centred or service delivery lens to inform the integration of care across the continuum. This review aimed to identify and summarise the available literature on lung cancer OCP.

**Methods:**

A scoping review was conducted, with literature across multiple databases and grey literature searched. Articles were included if the OCP was being used to manage adult patients with lung cancer and reported on either the development process and outcomes and/or barriers and facilitators associated with optimal care pathway development and/or uptake.

**Results:**

Of the 381 references screened, 32 articles were included. The lung cancer pathways reviewed varied significantly. A number of themes were identified including the development and implementation of the OCP; the use of quality indicators to audit the OCP; and studies on outcomes of the OCP incorporating timeliness of care delivery, patient experiences and health care utilisation and costs.

**Conclusions:**

The limited number of relevant articles found in this review may suggest that an OCP for lung cancer is still in its preliminary stages across the broader health systems.

## Introduction

Lung cancer is a significant cause of morbidity and the leading cause of cancer mortality in Australia [1]. In 2016, lung Cancer accounted for about 9% all new cancers diagnosed and 18% of all cancer deaths in Australia [1]. Limited data exists detailing how lung cancer patients flow through the healthcare system, and the delays and barriers that may interfere with the patient journey. The availability of evidence-based guidelines and multidisciplinary meetings has improved clinical effectiveness but does not automatically translate into improved quality of care [2]. Various studies done in Australia and other countries have revealed delays in lung cancer diagnosis and treatment, especially for rural patients [3,4,5,6,7]. The limited access to cancer care services is a significant issue faced by residents of rural, remote and regional communities [8,9,10].

Cancer patients utilise a wide range of services from multiple providers at various points during their cancer journey, including oncologists, primary care physicians, nurses, pharmacists, physiotherapists and social workers [6]. They frequently experience fragmented health care journeys and suffer from lack of continuity of care [11]. Given the high cost and complexity of their evolving needs from the point of diagnosis to either survivorship or palliative care, cancer patients require care that is integrated across providers and settings over time [12,13]. Implementation of a ‘Cancer Care Pathway’ might be a way of improving patients’ care experiences and to improve the efficiency of the health system [14].

Optimal care pathways are structured, multidisciplinary care plans for a specific clinical condition, which describe the tasks to be pursued, their timing, sequence and the professionals involved [15,16]. Implementation of optimal care pathways has been shown improve the outcomes of cancer patients, improve patient satisfaction and reduce costs [17]. Although the elements and frameworks are similar for many cancer-related pathways, each type of cancer requires specific approaches in terms of investigations and management modalities and applications; thus, requiring adaptations of pathways to both the kind of cancer and to local needs and settings [18]. Various international oncology societies have published criteria for the development and implementation of oncology pathway programs within their jurisdictions as listed in Table 1 [20,25,26,27,28,56]. Cancer Australia has published optimal care pathways for common cancers, including lung cancer, to guide the delivery of consistent, safe and high-quality care for cancer patients in Australia [20].

**Table 1:** Examples of optimal lung cancer pathways.

Group	Pathway	Year

Cancer Council, Australia [20]	Optimal cancer care pathways for people with Lung Cancer.	2016
Cancer Care Ontario, Canada [25]	Lung Cancer Pathway Map	2019
Swedish Lung Cancer Study Group, Sweden [26]	Recommendations from the Swedish Lung Cancer Study Group	1999
Lung Clinical Expert Group, UK [27]	National Optimal Lung Cancer Care Pathway implementation guide	2017
UK Lung Cancer Coalition, UK [28]	Implementing National Optimal Lung Cancer Pathway	2018
National Health Service (England) [57]	Implementing a timed Lung Cancer Diagnostic Pathway	2018

However, there is currently only limited consensus around the definition of the optimal care pathway, and there are various terminologies used interchangeably in the literature [19,21]. Little is known about how integrated care plans for cancer patients are developed, including the definition of core activities, the specifications for facilitators and the determination of indicators for assessing impact [22]. There is also only limited data to guide the implementation of optimal care pathways for lung cancer patients in the cancer centre environment. While major metropolitan cancer centres have the capacity to access all components of the optimal care pathway, including access to clinical trials, many regional centres may not have access to various components of the pathway [15]. This may mean that the patients may not receive the recommended investigations and care [23]. Due to the large geographical catchment area and the greater travel distances for patients, our regional cancer centre has embarked on developing cancer care pathways suitable for our region.

As part of developing our own lung cancer pathway to cater for our population, this review aims to identify and summarise the available literature on lung cancer pathways. A scoping review was conducted in order to explore the key components of lung cancer care pathways, identify the facilitators and barriers associated with their use and to examine the outcome measures that have been determined to assess their impact.

## Methodology

This review of the literature used the scoping review methodology as defined by Arksey and O’Malley [24]. Scoping reviews are commonly used to understand the existing breadth of research on a topic, identify gaps in the existing literature and assess the need for further research [24]. Arksey and O’Malley described five stages of methodological framework for a scoping review.

Our research question was refined to: ‘What is known from the existing literature about the implementation and evaluation of optimal care pathways for people with lung cancer?’

### Identifying relevant studies

Published articles were identified from electronic literature databases including MEDLINE, PubMed, Embase, and Google Scholar. Hand searching of reference lists and grey literature, including Cancer Institute publications, government websites, websites of cancer societies and cancer foundations was also done. The literature search was done using key search terms; (‘optimal care pathways’ or ‘integrated care pathways’ or ‘critical pathways’ or ‘clinical care pathways, protocol’) AND (‘lung cancer’ or ‘lung neoplasm’). Only articles in the English language were selected.

Two authors (ZO and AB) independently reviewed and screened the abstracts for selection, using the following inclusion criteria:

Articles were included if,

- The optimal care pathway was being used to manage adult patients with lung cancer.
- The article reported on either the development process and outcomes and/or barriers and facilitators associated with optimal care pathway development and/or uptake.

Articles were excluded if:

- The optimal care pathway was used for management of cancers other than lung cancer.
- The articles were concerned with only screening and prevention of lung cancer.
- The articles purely focusing on treatment algorithms of specific sub-specialities.

### Study selection

**Figure 1:**
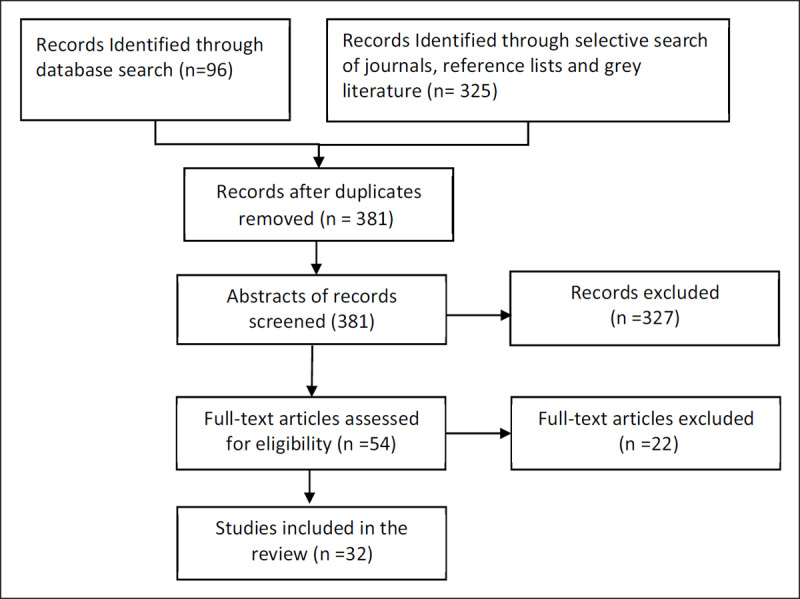
Flow chart for selection of articles.

The process of selection of articles for the final review is depicted in Figure 1.

Reasons for excluding full text articles included the following: articles dealing only with post-operative care (six), studies looking at systemic therapy in lung cancer (five), articles that included other types of cancers apart from lung cancer (four), articles dealing with molecular pathways (four) and articles that consisted of systematic review (three).

### Categorisation of studies into themes

Two authors (ZO, SS) independently reviewed the results and categorized the studies into sub-themes. Final themes were developed through consensus between authors and as follows:

- Development and implementation of lung cancer pathways
- Use of quality indicators to audit lung cancer care pathways
- Studies on outcomes of lung cancer pathways:
  - Improved timeliness of care delivery
  - Studies on patient experiences
  - Studies on health care utilisation and costs

### Charting the data

Data are summarised under each category in Table 2 (supplementary material).

## Results

A total of 421 references were retrieved and from these, 32 articles were selected for the final review. Out of these, six were guidelines published by professional organisations on implementing optimal care pathways for lung cancer, in their respective countries (Table 1). Eight of the articles dealt with development and implementation of optimal lung cancer care pathways. Five studies were on auditing lung cancer care pathways using quality indicators, four on evaluating the patients’ experience of lung cancer care pathways, five studies were on timeliness of care using lung cancer care pathways and four studies on health care utilisation and costs associated with lung cancer care pathways.

The care pathways vary significantly in their formats and implementations, depending upon the health system. Articles originated from a range of countries including both public and private health systems. Out of 32 articles, thirteen were from the United Kingdom, four were from the United States, four from Canada, three from Australia, four from Italy, and the remainder from other countries.

Various government organisations and professional bodies have published guidelines for implementation of optimal lung cancer pathways [20,25,26,27,28]. Although there are variations in the recommended investigations and timeframes, all the guidelines aim to provide timely and effective care for people with suspected lung cancer. These pathways chart the optimal journey of a person with suspected lung cancer from diagnosis to treatment and follow up. They also contain information on lung cancer prevention, supportive care and palliative care. A wide range of clinicians, peak health organisations, consumers and carers were consulted and participated in their development [20].

### Development and implementation of lung cancer care pathways

Eight studies focused mainly on the processes and challenges of implementing lung cancer care pathways. The implementation of lung cancer pathways was made in multiple steps, involving multi-disciplinary teams within the oncology community. One of the first steps in implementing a lung cancer pathway is ‘process mapping’, in which the existing lung cancer pathway in the region is mapped and weak points identified [28]. Streamlining administration and use of the best information technology tools are also an important part of implementing the pathway [29].

Most of the care pathways were designed by multidisciplinary teams, consisting of clinicians who are directly involved in lung cancer management and health care researchers [24,30,31,32,33,34,35,36]. Patient involvement in the design of care pathways was quite limited, with only a few studies reporting patient engagement [36,37]. The care pathways needed to be revised over time, and there were specific teams who were tasked with monitoring the uptake of the pathway [30,32,38,39,40]. The care pathways were often linked to patients’ electronic medical records [30,40,41]. By using computer-based platforms, aggregating data on treatment decisions and care delivery were facilitated [40]. There was a strong emphasis placed upon sharing information between teams and across organisations in most care pathways [31,39,40,41].

In the ‘Cancer Care Ontario Lung Cancer Disease Pathway’, diagnostic and treatment pathways for lung cancer were posted on a website [32]. Representatives from primary care, public health, occupational medicine, oncology, patients and their care givers participated in formulating this pathway [32]. Some centres addressed the delays in lung cancer care by redesigning the regional model of care [29]. The Ottawa Hospital Model operationalised the lung cancer diagnostic pathway and optimised patient flow from referral to initiation of treatment. All the stakeholders including health care professionals, patients, caregivers and administrators, participated in re-designing the lung pathway [29]. Lung cancer care pathways can be implemented in a regional setting, where the GPs have access to same-day chest X-ray and CT scan and adequate staffing of daily lung cancer clinics [42]. A lung cancer patient navigator coordinated patient referrals from a large rural catchment area [42].

Various modifications to the lung cancer care pathways have reduced delays to diagnosis and improved the efficacy of the pathways, including employment of a lung cancer nurse specialist [43,44], straight-to CT pathway [44] and immediate reporting of chest x-rays by a radiographer [45]. Quick turnaround time for pathology reporting is essential for implementing an optimal lung cancer pathway, and delays in pathology reporting can be due to logistical issues or requests for molecular testing [46]. Qualitative studies on local lung cancer care pathways in the National Health Service, England, showed that the strengths of the services included leadership, good team work, good relationships within the service, ownership, and quick turnaround times for some of the tests [47]. The challenges included radiology and oncology capacity, insufficient clinical information from GPs and lack of consistency and delay in Multi-Disciplinary Treatment decisions [47]. One-Stop Clinics, where the patients are seen by multiple specialists on the same day and multiple investigations are organised have also been successfully trialled [47].

### Using quality indicators to audit lung cancer care pathways

There were five studies on quality indicators and auditing lung cancer pathways [33,37,38,48,49]. The group from the University of Udine in Italy selected quality indicators from international guidelines and after discussions with the local multidisciplinary thoracic malignancy group [33]. These quality indicators can be used to identify problems in the local lung cancer pathway, such as delays and non-adherence to clinical guidelines [38]. Investigators from the University of Sheffield interviewed professionals from primary, secondary, tertiary and palliative care, and developed paper-based forms to monitor the progress of lung cancer patients and audit the key standards within the pathway [37]. Quality indicators can also be used to verify adherence to clinical practice guidelines and elicit some critical issues in the care of lung cancer patients [48]. The results from auditing studies need to be shared with hospital managers, with the aim of redesigning lung cancer care pathways and improving the efficiency of care [48].

### Outcomes of lung cancer care pathways

#### 1. Improved timeliness of care

Optimal lung cancer care pathways aim to reduce the time delays experienced by patients in seeing a specialist to do specific investigations and to start treatment [24,36]. Several studies have described the processes that were implemented in the respective hospitals to reduce the time to diagnosis and treatment [30,35,44,51,52]. This was done using novel management systems [30], and by the provision of adequate resources and improved communication between departments [35]. Quality improvement instruments like the ‘Lean Model’ programme was successfully utilised in order to reduce the delays in the lung cancer pathway and to improve patient flow [30]. Information technology-driven optimal care pathways and virtual clinics shortened diagnostic timeframes, even in regional hospitals [52]. One of the methods used to reduce delays in diagnosis of lung cancer is to automatically trigger a referral for CT scan if the chest x-ray is abnormal [44].

#### 2. Effect on health care utilisation and costs

Standardised clinical care pathways for the investigation of patients with lung cancer allow for a reduction in the time interval between suspicion of lung cancer and treatment, lower costs and increase patient satisfaction and quality of care [52]. Treatment pathways mainly deal with the best systemic treatment options for a lung cancer patient at a certain point of care. Systemic therapy was selected based on its efficacy, toxicity profile and cost-effectiveness [31,39,40]. Pathways reduced the cost of lung cancer treatment without compromising survival [39,40].

#### 3. Studies on patient experience

One of the aims of implementing an optimal lung cancer care pathway is to improve patients’ experiences and satisfaction with their care [20]. Perspectives of lung cancer patients, evaluated using the OPTION (Opportunity for Treatment in Oncology) questionnaire, give the highest scores to ‘respect’, ‘satisfaction’, and ‘trust’ [53]. Offering written communication about diagnosis and maintaining patient privacy during consultations improves patient satisfaction [54]. Hagglund et al. (2015) from the Karolinska institute in Sweden, studied patients’ experiences of living with lung cancer and created a ‘patient journey model’ [34]. The problems experienced by patients in each phase were identified and eHealth solutions for these problems were proposed [34]. Investigators from the University of Memphis compared patients’ perspectives of multidisciplinary lung cancer care to routine serial care [36]. They found that participants preferred multiple physicians working together as a team to decide on the best plan of care.

### Barriers to implementation of optimal lung cancer care pathways

Barriers to implementing lung cancer care pathways can occur at various levels. Some of the common challenges include resource limitation, diagnostic and treatment capacity and complex patients [47,55]. Some hospitals will not have specialist investigations like PET scans or endobronchial ultrasounds and have to refer patients to other centres, thereby delaying the diagnosis and staging of lung cancer [55]. There will be limitations to the number of CT scans or biopsies that can be done in a day. Following the initiation of a new lung cancer pathway, there may be an increased number of primary care requests for CT scans [47]. Collaborative working and good communication between various departments can overcome these issues [28]. Clinicians can be reluctant to use new forms or pathways [30,37]. Two common barriers to implementing the pathway in the cancer care Ontario study were slow referral processes and lack of administrative support [32]. Members of the lung cancer pathway team would have concerns about over burden and increased workload [28]. Part-time GPs and lack of ability to review X-ray and CT reports on a daily basis can pose a challenge [28]. Local lung cancer pathway data has to be validated for quality control, but this could be a challenge [28]. Some of the health services would have difficulty in funding the optimal lung cancer pathway implementation [56,57].

## Discussion

Optimal cancer care pathways map the cancer patient’s journey for specific tumour types in order to promote quality cancer care and improve the patient’s experiences. The optimal care pathway for lung cancer has been endorsed by health authorities in Australia and its key features include the following: patient-centred care, safe and quality care, multidisciplinary care, and improved coordination and communication [20]. This pathway outlines seven critical steps in a lung cancer patient’s journey which are prevention, initial investigations and referral, diagnosis and treatment planning, treatment, follow up after treatment completion, management of recurrent cancer and end-of-life care [20]. Similar lung cancer care pathways have been developed in other countries [25,26,27,28]. Previous reviews on optimal lung cancer care pathways were focused on timeliness of care [56,58] or barriers to early diagnosis [59]. There are no overviews of the whole lung cancer care pathway from prevention to end of life care. In addition, components of the pathways are likely to be context specific based on many factors including resources. Therefore, a one size fit all approach is not applicable in this setting and contents need to be adapted for local needs and based on resources. One of the aims of this scoping review is to identify existing successful examples and innovations in the literature so that centres wanting to establish local lung cancer pathways can leverage existing experience.

The type and scope of lung cancer pathways in this review varied significantly. The studies included in this review were either related to implementing national optimal cancer care pathways in their institution or auditing the quality of the optimal care pathway. Some lung cancer pathways were employed in single hospitals while others were employed on a much wider scale; focusing exclusively on the clinical elements of care or have targeted individual stages in the cancer trajectory, rather than considering integrated care along the continuum [11,12].

Implementation of lung cancer care pathways had positive impacts on patient care in most of the studies. It has shown to reduce waiting times for diagnosis and treatment [30,35,44,50,51]. This appears to be associated with better patient outcomes and higher patient satisfaction [34,36,52,53,54]. The cost of cancer therapies is a significant issue, and it has been rising with the introduction of new (and often more expensive) therapies [13]. Studies looking at the cost effectiveness of lung cancer pathways demonstrated reduced costs [39,40,52]. Cost savings were made either by selecting cheaper, but appropriate chemotherapy agents [39,40] or by reducing delays and avoiding unnecessary investigation [52].

This review has identified some strategies for implementation of various steps of lung cancer care pathways. Initial steps include engagement of multidisciplinary professionals and ‘process mapping’. Process mapping identifies components of existing systems and areas of deficiencies [22]. The engagement and involvement of clinical providers helps foster a sense of ownership [2,23,24]. Development and implementation of the lung cancer pathway should be driven by clinicians, health scientists and consumers [31] although most of the studies in this review did not have formal mechanisms for involving consumers in design.

Some examples of structural factors that enable implementation include a dedicated team of professionals to oversee the implementation and operationalisation of the care pathway and undertake quality improvement activities. [4,24]. Many centres employed dedicated lung cancer care nurses or nurse navigators or dedicated physicians to coordinate and spearhead their lung cancer services [42,43]. Cultural factors include leadership, teamwork, and good relationships within the service as exemplified by the ACE Lung Cancer Pathway cluster in United Kingdom [47].

Our review also identified some innovations and examples that were used to streamline and expedite the patient journey at the diagnosis stage of the pathway. These include the following: access by GPs to same day CXR and CT scans (42), straight-to CT pathway (44), automatic referral for CTs when an abnormality was found on CXRs (44), and immediate reporting of CXR by radiographers. In terms of management and multidisciplinary stage, One-Stop Clinics, where the patients are seen by multiple specialists on the same day and multiple investigations are organised have also been successfully trialled [25]. However, this may not be feasible at smaller regional or rural centres due to workforce and space issues.

In terms of patient information management, electronic and web-based platforms seem to be more widely used to implement lung cancer pathways [5,23,24,26]. With the widespread use of smartphones and tablets, these pathways may be accessible to most specialist and primary care providers and patients. Information technology-driven optimal care pathways and virtual clinics shortened diagnostic timeframes even in regional hospitals [14]. Electronic systems offer other advantages including ease of remote access, data sharing by MDT members and use of quality indicators to monitor and improve the Pathway [60].

Some of the studies in this review have utilized quality indicators that were used by other centres for research on lung cancer pathways [4,27,28,29]. These quality indicators can be used to identify problems in the local lung cancer pathway, such as delays and non-adherence to clinical guidelines [4]. A good practice is to share these data with managers with the aim of improving lung cancer care pathways and the efficiency of health care delivery [29]. The pathway tools will not achieve maximum success without an actual engagement of the hospital management [33]. Even after the cancer care pathway is implemented, it must be audited periodically, in order to ensure that all aspects of pathway are running optimally [38,49]. This requires a dedicated team of professionals, who can periodically allocate time to hold meetings and monitor the performance of the care pathways, and continuously adjust and improve their content [38].

Despite investment by governments on lung cancer pathways, clinicians, patients and system managers face many barriers to implementing the lung cancer pathways. Some example of barriers include the following: inadequate support from clinicians because of concerns of increased-workload (28), limited integration between primary care and tertiary care (47), slow referral processes, limited resources and administrative support, lack of training for providers regarding the pathway and inadequate information technology support (32). While larger teaching hospitals have put significant resources into fast tracking diagnosis, diagnostic and staging technologies and molecular genotyping of lung cancer [6], this may not be possible in the community setting and smaller centres due to limitations of resource, workforce and leadership capabilities.

Our review has some limitations. To manage scope, we excluded studies examining the pathways in other cancers that might have solutions applicable to lung cancer. We identified only a few studies on implementing care pathways in predominantly rural populations that face many of workforce and resource related barriers [4]. However, rural centres wishing to set up lung cancer pathways could take advantage of telehealth technologies to connect to larger centres and acquire workforce capabilities remotely. In addition, these centres could leverage consumers and the data on inequity of access and disparity in outcomes to lobby for additional resources to set up care pathways.

## Conclusion

The limited number of relevant articles found in this review may suggest that an optimal care pathway for lung cancer is still in its preliminary stages across the broader health systems. Clinical leadership, involvement of multidisciplinary clinicians and managers, dedicated administration or nursing support, innovative solutions, integration of pathways into user-friendly IT platform, and quality improvement mechanisms seem to be important components for successful implementation of a lung cancer care pathway. Smaller centres could take advantage of telehealth technologies to connect to larger centres and acquire workforce capabilities remotely. Depending on the geographical location and resource constraints, the optimal lung cancer pathway needs to be adapted for local setting with inclusion of innovations to overcome deficiencies. Since different health services have different types of referral patterns for lung cancer, local workflow issues must be considered and problems with the lung cancer pathways need to be solved at the local health service level.

## Additional File

The additional file for this article can be found as follows:

10.5334/ijic.5438.s1 Appendix Table 2. Table of themes and related studies.
