# Supplementary material for: Optimal Care Pathways for People with Lung Cancer- a Scoping Review of the Literature
Source: Int J Integr Care. 2020 Sep 28;20(3):14. doi: 10.5334/ijic.5438 (PMC7528692; doi:10.5334/ijic.5438)
Supplement: Appendix Table 2. — Table of themes and related studies. [file ijic-20-3-5438-s1.pdf]

Appendix

Table 2: Table of themes and related studies

**Author, year of publication, country**	**Design**	**Setting and sample size**	**Description of the study**	**Results**	**Conclusion of authors**
**Studies on developing and implementation of lung cancer pathways**					
Evans WK, Ung YC, Assouad N, Chyjek A, Sawka C 2013, Canada	Prospective Implementation study	Large public health care system- urban & rural.	Cancer care Ontario lung cancer disease pathway which formulated evidence-based guidelines for prevention, screening, diagnosis, treatment, palliative care and survivorship during the lung cancer patient's management. Multidisciplinary, focus-group meetings held to develop quality improvement projects for lung cancer pathway.	Standardised diagnostic and treatment pathways for lung cancer was developed.	Regional cancer centres can undertake adequate quality improvement initiatives

**Author, year of publication, country**	**Design**	**Setting and sample size**	**Description of the study**	**Results**	**Conclusion of authors**
Fung-Kee-Fung M, Maziak DE, Pantarotto JR, Smylie J, Taylor L, Timlin T, et al. 2018, Canada	Prospective implementation study	University hospital.	Regional process redesign of lung cancer care by lean optimization of diagnostic procedures and patient education including coordinated referral review and integrated navigation.	Twelve major processes in referral, review, diagnostics, assessment, triage, and consult were redesigned. The Ottawa Hospital now provides a diagnosis to 80% of referrals within 28 days. The median patient journey from referral to initial treatment decreased by 48%, from 92 to 47 days.	The initiative optimized regional integration from referral to initial treatment. Transformation initiatives across the continuum of care are needed to incorporate best practice and optimize delivery systems for regional populations.

Author, year of publication, country	Design	Setting and sample size	Description of the study	Results	Conclusion of authors
Manley S, Delaney L 2015 Australia	Descriptive study	Rural population.	GP can access to same day chest radiograph and CT scan. Daily lung cancer clinics were set up, staffed by dedicated consultants. A non-clinical patient navigator was appointed to coordinate patient flow in the new pathway.	Developing a lung cancer referral and diagnostic pathway in a regional setting.	N/A

Author, year of publication, country	Design	Setting and sample size	Description of the study	Results	Conclusion of authors
Bennett A, Patrick C, 2019 UK	Retrospective audit.	113	An audit of value of the lung cancer nurse specialist in reducing pathway timings in the local optimal lung cancer pathway. The Cancer Nurse Specialist being notified of abnormal Chest X-rays requested by the GP, contacting the patient and notifying the GP. They then request a CT scan and if abnormal refer in for a 2-week outpatient appointment with a respiratory specialist.	During that period 113 abnormal CXRs were flagged to the Cancer Nurse Specialist. Of these 53 patients had normal CT scans and discharged back to the GP. 60 patients were diagnosed with lung cancer. In the previous year 320 patients were referred for 2WW OPAs with only 67 diagnoses.	With the introduction of the pathway, a significant number of unnecessary outpatient appointments have been avoided.

Author, year of publication, country	Design	Setting and sample size	Description of the study	Results	Conclusion of authors
Donitz N et al. 2017 UK	Randomised trial	Urban hospital 8732 patients	Impact of radiographer immediate reporting of chest x-rays from general practice on the lung cancer pathway. Half of the sessions per week (5) were randomised to an immediate or routine chest x-ray (CXR) report by a reporting radiographer, with an immediate CT where indicated. Time taken to diagnosis of lung cancer or discharge from the lung cancer pathway was determined and Mann-Whitney test used.	Time to diagnosis of lung cancer for patients in the immediate arm was a median of 21.5 days compared with 30 days in the routine CXR arm ($p=0.012$). For patients with a suspicious CXR, diagnosis of lung cancer was achieved in a median of 18 days compared with 32 days in the routine arm ($p=0.0375$).	Immediate reporting of CXRs referred from primary care by radiographers reduced time to diagnosis of lung cancer by a median of 14 days.

Author, year of publication, country	Design	Setting and sample size	Description of the study	Results	Conclusion of authors
Lloyd KL, Rice A, Robert's JL, Brambilla C, Poppet S, Kemp S, et al. 2019 UK	Retrospective audit.	40	Retrospective data collection from a 3-month period on turnaround times of pathology diagnostic reports of lung cancer.	60% of cases had diagnosis reports available within 1–3 days, with all available within 9 calendar days. Delays were either due to logistics (transport, delays in antibody replacement), or molecular requesting occurring post-diagnosis.	The results of the retrospective data analysis have led to the introduction of a standardized molecular request form.
Fuller L, Robson S, Tasker C. 2017 UK	Descriptive study	National health service	Implementing a One-Stop Lung Cancer Clinic. Following a CT scan, diagnostic tests are being combined so that patients can have multiple diagnostic tests in one day.	Implementation of One-Stop clinic led to improved chances of achieving the 62-day pathway timeline.	Needs coordinated effort from all the staff.

**Author, year of publication, country**	**Design**	**Setting and sample size**	**Description of the study**	**Results**	**Conclusion of authors**
Gill B 2016 UK	Qualitative survey	20	Lung Cancer Pathways Cluster Qualitative Research Results by Cancer Research UK. The telephone interviews were conducted to gather qualitative information as well as information about pathway configurations.	Strengths of the local lung cancer pathways included leadership, teamwork, good relationship among team members, strong patient tracking arrangements and quick turnaround times for some tests. Challenges faced by the local pathways included inadequate capacity for radiology and pathology, insufficient clinical information in GP referrals and waiting time for MDT decision.	Concerted effort by the local teams can achieve significant positive change.
**Studies on use of quality indicators in Lung Cancer care pathways**					

Author, year of publication, country	Design	Setting and sample size	Description of the study	Results	Conclusion of authors
Fasola G, Menis J, Follador A, De Carlo E, Valent F, Aresu G, et al 2018 Italy	Retrospective audit and analysis	University hospital. *169.*	Quality of care of lung cancer patients was measured through fifteen indicators.	Eight of fifteen indicators were not in line with the benchmarks.	Integrated care pathways confirm to be feasible and to be an effective practical tool. Periodic measurement of quality indicators is necessary to ensure clinical governance of patient pathways.
Fasola G, Rizzato S, Merlo V, Aita M, Ceschia T, Giacomuzzi F, et al. 2012 Italy	Retrospective audit and analysis	University hospital 175.	Flowcharts were drawn for management of early, locally advanced and metastatic lung cancer disease respectively. Evaluated the lung pathway using 11 quality indicators to identify problem areas.	Diagnostic workup, multidisciplinary team care and medical treatment of advanced disease were areas of good performance. Management of early stage lung cancer needed improvement.	Auditing the process of caring for non-small cell lung cancer patients is feasible and offers room for improvement.

**Author, year of publication, country**	**Design**	**Setting and sample size**	**Description of the study**	**Results**	**Conclusion of authors**
Rizzato S, Merlo V, Aita M, Sibau A, Menis J, Gurrieri L, et al 2011 Italy	Retrospective audit and analysis	University hospital 175	A multidisciplinary quality improvement project on assessing quality of existing Lung Cancer Pathway.	Eleven quality indicators were identified to assess clinical, organizational and economic aspects of patient care.	By means of a limited set of quality indicators, adherence to clinical guidelines in the care of lung cancer patients could be monitored. Results need to be shared and discussed with Hospital Managers, with the aim of guiding the redesign of Integrated care pathway.

**Author, year of publication, country**	**Design**	**Setting and sample size**	**Description of the study**	**Results**	**Conclusion of authors**
Kaltenthaler E, McDonnell A, Peters J. 2001, UK	Prospective implementation study	University hospital (urban) 53	Development of paper-based forms to monitor the progress of lung cancer patients and auditing key standards within the pathway. Three stages: i) development of a paper version of the form for each point of care on the lung cancer pathway; ii) trial period of use; and iii) evaluation of the paper form in terms of its success as a tool to capture appropriate data.	Completed preclinical record forms were available for 68% of patients with 42% of these patients also having a record of the decisions made at the multidisciplinary team meeting. Completion of forms that covered the later stages of the care pathway was limited.	The forms were acceptable to users and provided accurate information at key points on the patients' journey in the lung cancer pathway.

Author, year of publication, country	Design	Setting and sample size	Description of the study	Results	Conclusion of authors
Kim M L, Matheson L , Garrard B, 2018 Australia [49]	Retrospective review	Regional cancer centre	Use of clinical quality indicators to improve lung cancer care in a regional/rural network of health services	The multidisciplinary lung cancer clinic has streamlined the access to lung cancer services, including specialist consultations, diagnostics and therapeutic services, in a regional setting.	The regular monitoring of clinical quality indicators serves as a useful method of quality assurance in the care of patients with lung cancer.
**Studies evaluating patient experience of Lung Cancer Care Pathways**					

**Author, year of publication, country**	**Design**	**Setting and sample size**	**Description of the study**	**Results**	**Conclusion of authors**
Bravi F, Ruscio ED, Frassoldati A, Cavallesco GN, Valpiani G, Ferrozzi A, et al 2018 Italy	Cross-sectional study.	77 patients and 38 health professionals.	Opportunity for Treatment in Oncology (OPTION) questionnaires were administered to 77 patients, and the Care Process Self-Evaluation Tool (CPSET) questionnaires were given to 38 health care professionals.	In an Integrated Care Plan, the views of patients and health care professionals overlap on aspects considered important, namely, a person-centered approach. Their perception of weaknesses is also similar a relative lack of patient communication and cooperation between hospital staff and GPs.	The lung cancer pathway is a patient-centered intervention that enables care to be shaped for patient needs in order to improve the quality and efficiency of service and clinical outcome.
Hagglund M, Bolin P, Koch S 2015 Sweden	Descriptive Qualitative study	University hospital 9 patients with lung cancer	Describes the lung cancer care process as experienced by the patients and analyses the problems they encounter throughout the journey. Focus group interviews of patients.	Identified problems faced by lung cancer patients.	Patient journey models and qualitative analysis of patients' experiences are powerful tools that can be used to improve health care.

Author, year of publication, country	Design	Setting and sample size	Description of the study	Results	Conclusion of authors
Kedia SK, Ward KD, Digney SA, Jackson BM, Nellum AL, McHugh L, et al 2015 USA	Qualitative evaluation study	22 patients and 24 care givers	A 'one-stop shop' model of care was tried, where all the concerned specialists reviewed patients at the same time. Focus group interviews of patients and caregivers comparing multidisciplinary model of lung cancer care with serial model of care.	Multidisciplinary care improved physician collaboration, patient-physician communication and patient convenience, while reducing redundancy in testing, Improved coordination decreased confusion, stress and anxiety. Negative experience of serial care included poor communication among physicians, insensitive communication about illness, delays in diagnosis and treatment, misdiagnosis and mistreatment.	Multidisciplinary care was perceived as more patient-centered, effective, safe, and efficient than standard serial care. It was also believed to improve the timeliness of care and equitable access to high quality care.

Author, year of publication, country	Design	Setting and sample size	Description of the study	Results	Conclusion of authors
McDowell G<br>2014<br>UK	Patient experience questionnaire	National health service	Patient experience questionnaire about Optimal lung cancer care Pathway was distributed in the outpatient setting within The Beatson Oncology Centre over a period of 6 weeks.	90% Felt their doctors were sensitive in breaking bad news<br>71% Completely understood the purpose of investigations<br>Nearly 50% had not been offered written information on lung cancer.<br>94% Felt there was enough privacy during a consultation.<br>80% Felt their emotional needs were met throughout the investigation & treatment process.<br>93% were given contact information of cancer specialist nurse or support network	The questionnaire highlights the excellent quality and quantity of communication supplied throughout the diagnosis and treatment process.<br>Adequately addressing both the emotional and physical needs in most patients.

Author, year of publication, country	Design	Setting and sample size	Description of the study	Results	Conclusion of authors
**Studies on** Improving timeliness of care					
Bakewell F, Hodgkiss M, Perumpalath B, Wright-Morris D, Severn H, Anwar S 2019 UK	Retrospective cohort study	452	The patient cohort was defined as those referred on a 2ww pathway due to an abnormal CXR at NUH between 03/07/2017 and 30/06/2018. We retrospectively reviewed the time from CXR to CT and the time from 2ww referral to decision to treat (DTT), comparing patients on the new 'straight to CT' pathway with those on the conventional pathway.	Of those 189 (41.8%) underwent a CT on the conventional pathway and 242 (53.5%) on the new pathway. Median time from CXR date to CT report was 12 days on the conventional pathway and 7 days on the new pathway. Of the eligible cohort, 135 patients (29.9%) were diagnosed with lung cancer: 18.7% on the conventional pathway and 39.5% on the new pathway. Median time from 2ww referral to DTT was 42 days on the conventional pathway and 36 days on the new pathway.	Implementation of a 'straight to CT' pathway has led to a reduction in the time to CT and Decision to Treat.

Author, year of publication, country	Design	Setting and sample size	Description of the study	Results	Conclusion of authors
Moneke J, Khan S, Hussain I. 2019 UK	Retrospective study	University hospital. 106+ 86	Patients who were diagnosed with lung cancer within the time period of January 2018 to February 2018 (n=86) were used as a control and compared to those in May to June 2018 (n=106).	Referral start date to diagnosis showed a significant improvement falling from an average of 51 days in the January–February 2018 period to 19 days in the May 2018 period. The diagnosis to treatment and the referral start date to treatment stages, showed a similar trend falling from 24 days to 11 days and 65 days to 25 days respectively.	The findings have shown a significant reduction in waiting times across most stages of the patient referral pathway within the post-implementation period

**Author, year of publication, country**	**Design**	**Setting and sample size**	**Description of the study**	**Results**	**Conclusion of authors**
Kutubudin F, Robinson R, Deus P, Hughes K, Wight A 2017 UK	Implementation study	Regional patients 210	IT-driven lung cancer pathway, linking primary care with secondary care radiology, respiratory and outpatient booking services (including endoscopy). A daily consultant virtual clinic. All further diagnostic investigations are pre-arranged	Time from virtual review to formal cancer clinic - mean 5.8 days (target 5 days). Time Flagged CXR to MDT discussion (target 21 days)- 23.9 days. patients informed of treatment plan within 28 days	Diagnostic timeframe has been shortened by average of 15 days. 28- day target is achievable even outside tertiary centres.
Aasebo U, Strom HH, Postmyr M 2012 Norway	Retrospective cohort study.	University hospital 69	Study of workup times for patients with lung cancer using the “Lean” quality improvement process (using mechanisms to identify and sustain high-value encounters and eliminate obstacles) to improve patient flow.	Decrease in the workup time from a mean of 64 days to 16 days, and the median time from diagnosis to surgery was reduced from 26.5 days to 15 days.	It is feasible to improve patient flow for patients with lung cancer by employing the Lean method as a pathway instrument.

Author, year of publication, country	Design	Setting and sample size	Description of the study	Results	Conclusion of authors
Jiang T, Ren S, Li X, Su C, Zhou C, O'Brien M<br>2017<br>China	Retrospective analysis	4000 patients in University hospital	Fast-track diagnostic pathway for lung cancer patients.	In this pathway, the median time for initial respiratory consultation to treatment decision making was only 4 days.	A rapid diagnostic system for lung cancer needs following factors- centralization of resources; fully committed specialists, a daily MDT ,molecular detection laboratory with; a coordinator to monitor the whole; capacity to keep patients in the hospital , an electronic system of real- time communications and reports of the results.
**Studies on health care utilisation and costs**					

Author, year of publication, country	Design	Setting and sample size	Description of the study	Results	Conclusion of authors
Jackman DM, Zhang Y, Dalby C, Nguyen T, Nagle J, Lydon CA, et al. 2017 USA	Cost-effectiveness analysis	370 University hospital	Study comparing cost effectiveness of the Dana-Farber lung cancer pathway, which is a web-based platform for real-time decision making on and data collection on systemic therapy.	The total 12-month cost of care demonstrated a $15,013 savings after the implementation of pathways. Clinical outcomes were not compromised, with similar median overall survival times (10.7 months before v 11.2 months after pathways; P = .08).	After introduction of a clinical pathway in metastatic NSCLC, cost of care decreased significantly, with no compromise in survival.

Author, year of publication, country	Design	Setting and sample size	Description of the study	Results	Conclusion of authors
Neubauer MA, Hoverman JR, Kolodziej M, Reisman L, Gruschkus SK, Hoang S, et al 2010 USA	Cost-effectiveness analysis	1409 Community oncology network	Electronic pathway incorporating expert recommendations for systemic therapy for various stages and lines of non-small cell lung cancer patients in the outpatient community setting. Study comparing cost-effectiveness of treating patients with chemotherapy, on or off the pathway.	The average cost of care for patients on the pathway was 35% less compared with that of patients treated off the pathway. Patient survival was not affected.	treating patients according to evidence-based guidelines is a cost-effective strategy for delivering care to those with NSCLC.

**Author, year of publication, country**	**Design**	**Setting and sample size**	**Description of the study**	**Results**	**Conclusion of authors**
Ellis PG 2013 USA	Retrospective audit of pathway compliance	University cancer centre	Interactive software that allows for real-time decision-making Pathways for best treatment option. Regularly updated by expert committee of clinicians. Retrospective audit of compliance.	75% of non-small cell lung cancer patients treated according to the pathway.	Physician involvement is integral to a successful clinical pathways program. When appropriately developed and implemented, clinical pathways are an effective tool for standardizing care and ensuring quality.

Author, year of publication, country	Design	Setting and sample size	Description of the study	Results	Conclusion of authors
Shamji FM, Deslauriers J 2013 Canada	review	N/A	Review on benefits of Fast-tracking investigation and staging of patients with lung cancer	Standardized clinical care pathways for the investigation of patients with lung cancer allow for a reduction in the time interval between suspicion of lung cancer and treatment, lower costs, increased patient satisfaction, and quality of care.	Reducing delays for reference, investigation, and treatment through the use of standardized clinical care pathways may not improve overall long-term survival by much, but it has been well demonstrated that such a strategy is essential to improve quality of care and reduce costs through more consistent and efficient use of resources.
